# StarSeeker: an automated tool for mature duplex microRNA sequence identification based on secondary structure modeling of precursor molecule

**DOI:** 10.1186/s40709-018-0081-7

**Published:** 2018-06-15

**Authors:** Paschalis Natsidis, Ilias Kappas, Wojciech M. Karlowski

**Affiliations:** 1 Department of Genetics, Development & Molecular Biology, School of Biology, Aristotle University of Thessaloniki, 54124 Thessaloniki, Greece; 2 Department of Computational Biology, Institute of Molecular Biology and Biotechnology, Faculty of Biology, Adam Mickiewicz University, 61-614 Poznan, Poland; 3 Present Address: School of Medicine, University of Crete, Voutes University Campus, 70013 Heraklion, Crete, Greece

**Keywords:** miRNA maturation, Sequence prediction, Transcription regulation, Plant transcriptome

## Abstract

**Background:**

MicroRNAs (miRNAs) are small, non-coding RNA molecules that play a key role in gene regulation in both plants and animals. MicroRNA biogenesis involves the enzymatic processing of a primary RNA transcript. The final step is the production of a duplex molecule, often designated as miRNA:miRNA*, that will yield a functional miRNA by separation of the two strands. This miRNA will be incorporated into the RNA-induced silencing complex, which subsequently will bind to its target mRNA in order to suppress its expression. The analysis of miRNAs is still a developing area for computational biology with many open questions regarding the structure and function of this important class of molecules. Here, we present StarSeeker, a simple tool that outputs the putative miRNA* sequence given the precursor and the mature sequences.

**Results:**

We evaluated StarSeeker using a dataset consisting of all plant sequences available in miRBase (6992 precursor sequences and 8496 mature sequences). The program returned a total of 15,468 predicted miRNA* sequences. Of these, 2650 sequences were matched to annotated miRNAs (~ 90% of the miRBase-annotated sequences). The remaining predictions could not be verified, mainly because they do not comply with the rule requiring the two overhanging nucleotides in the duplex molecule.

**Conclusions:**

The expression pattern of some miRNAs in plants can be altered under various abiotic stress conditions. Potential miRNA* molecules that do not degrade can thus be detected and also discovered in high-throughput sequencing data, helping us to understand their role in gene regulation.

## Background

MicroRNAs are small, non-coding RNA molecules that play a key role in post-transcriptional gene regulation in plants, animals and some viruses. They are usually 21–24 nucleotides long and regulate a diversity of cellular processes such as growth, development, differentiation and apoptosis. In mammals, microRNAs regulate over 60% of the protein-coding genes [1].

MicroRNAs are produced through enzymatic processing of a primary RNA transcript, which can originate either from its own gene, usually found in intergenic regions across the whole genome, or from an intron of a protein-coding gene [2]. This transcript is called primary miRNA (pri-miRNA) and it is processed into a ~ 70 nucleotide-long precursor miRNA (pre-miRNA) by the enzyme Drosha. This process takes place inside the nucleus and the product is exported to the cytoplasm by a complex of Exportin-5 and Ran-GTP. Then, the pre-miRNA molecule is further cleaved into a ~ 22 nucleotide-long dsRNA by the RNase III enzyme Dicer [3]. This RNA is the miRNA:miRNA* duplex and it will give the functional miRNA by separation of the two clones. This miRNA will be incorporated into the RNA-induced silencing complex (RISC), which subsequently will bind to its target mRNA in order to suppress its expression.

**Fig. 1:**
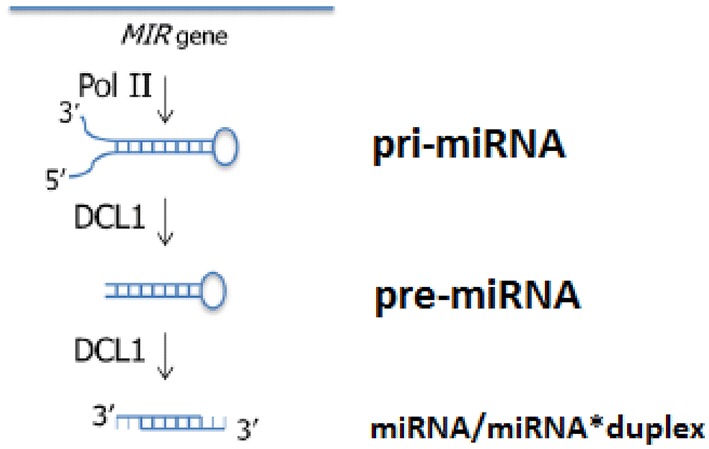
Part of miRNA biogenesis route in plants. The main difference from animal biogenesis is that in plants, the two cleavages are performed by the same enzyme (DCL1) inside the nucleus instead of two different enzymes (Drosha, Dicer), one inside and one outside the nucleus. The two hanging nucleotides in each side of the duplex can be seen. After this process, the duplex exits the nucleus and separates

In plants, miRNA biogenesis differs from the equivalent process in animals (Fig. 1). The main difference is that, in plants, both nucleolytic processes are taking place inside the nucleus and are being performed by the same enzyme, called Dicer-like1 (DCL1) [4]. Also, the created duplex is methylated by Hua-Enhancer1 (HEN1) to protect it against endonucleases, and it is then exported to the cytoplasm by an Exportin-5 homolog, called Hasty (HST). Following export, the duplex is disassembled and the mature miRNA is loaded on RISC, just as it happens in animals.

There are also some Drosha/Dicer independent biogenesis paths that involve the production of mature miRNAs from intronic sequences. This subset of intronic miRNAs are called *mirtrons* and lack the elements responsible for the recruitment of Drosha or any similar enzyme.

The fate of the miRNA* strand is usually degradation. However, some cases have been reported where both strands of the duplex become functional and participate in the mRNA silencing process. For example, in some cases the miRNA* strand of human miR-146a can produce two mature miRNAs, each of them targeting different genes contributing to thyroid cancer [5]. It has also been shown that in *Arabidopsis thaliana* the small RNA expression pattern changes under stress conditions. There is a possibility that some miRNAs*, which in normal conditions get degraded, undergo unique upregulation when presented to heat stress conditions [6].

**Fig. 2:**
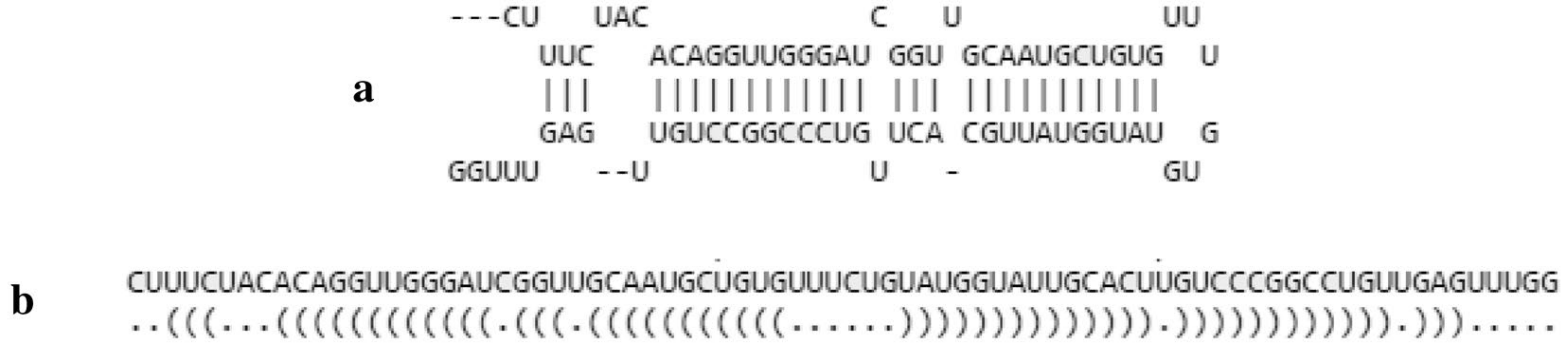
**a** The secondary structure of a pre-miRNA molecule. The gaps in base-pairing are represented by the “dash” character. Mismatches in the stem part that lead to unpaired bases can be seen. The loop is shown at the rightmost part of the molecule. **b** The first line represents the primary structure of the pri-miRNA shown in **a** and the second line indicates the “dot-bracket notation” of the same molecule

The secondary structure of the precursor molecule contains a hairpin with stem and loop parts. Some nucleotides in the stem can be unpaired forming internal loops or bulges. This secondary structure can be depicted by using the “dot-bracket notation” [7]. A full set of secondary structure, primary sequence and dot-bracket notation of a pre-miRNA molecule is shown in Fig. 2.

Analyzing miRNA structures and functions is a relatively new field of study in computational biology. Both experimental and computational approaches have been used to identify miRNAs and their target genes [8]. A complete list of the discovered and annotated miRNAs and their targets can be found in miRBase (http://www.mirbase.org) [9]. This database contains extensive data for every model-organism (human, mouse, fly, worm, *Arabidopsis* sp.) as well as for other organisms and it is regularly updated.

Several tools have been developed for the prediction of miRNA:miRNA* duplexes given a precursor sequence. MiRdup [10] uses a random forest classifier trained with miRbase data, while MiRPara [11] and MiRduplexSVM [12] use SVM classifiers to predict most probable miRNA coding regions in genome scale sequences and miRNA duplex given its hairpin sequence respectively, with the latter outperforming all to-date similar tools. MatureBayes [13] uses a Naive Bayes classifier to predict mature miRNA sequences from precursors. Other software, such as RNA-hybrid [14], are used to predict potential binding sites of miRNAs in large target RNAs.

The goal of the present work is to design a useful computational tool, named StarSeeker, that will predict the sequence of the miRNA:miRNA* duplex based on the structure of the precursor molecule. StarSeeker is a comprehensive and easy-to-use computational tool that will extract all potential miRNA* sequences, with respect to the two overhang nucleotides rule. It requires as input a list of precursor hairpin sequences, as well as a list of any known mature miRNAs that exist within these precursors. Using a simple algorithm based on precursor-mature miRNA matching and the secondary structure of the pre-miRNA, it returns a list with all possible miRNA* sequences that exist in the input hairpins.

## Methods

Our main approach was to develop a tool that outputs the miRNA* sequence, given the precursor and the mature sequences. The idea behind this algorithm was to use the property of the DCL1 enzyme leaving two nucleotide end overhangs during formation of the miRNA/miRNA* duplex.

We designed a software called “StarSeeker” which requires two files as input: one file that contains all the precursor sequences and another file that contains all the mature sequences. StarSeeker is implemented in Python. All the sequences must be provided in FASTA format. The program parses the input and creates an entry for each sequence using the BioPython SeqIO module (http://biopython.org/wiki/Main_Page). After this, every mature miRNA sequence is used as a query to search for matches within the precursor sequences dataset, creating precursor-mature pairs for each match found. At this stage, all existing entries have the following form:

(‘ath-MIR156a’,

‘CAAGAGAAACGCAAAGAAACUGACAGAAGAGAGUGAGCACACAAAGGCAAUUUGCA

UAUCAUUGCACUUGCUUCUCUUGCGUGCUCACUGCUCUUUCUGUCAGAUUCCGGUG

CUGAUCUCUUU’, ‘GCUCACUGCUCUUUCUGUCAGA’)

Duplicate entries are deleted, so the final dataset is non-redundant. However, mature sequences are allowed to match with more than one precursor, and precursors are allowed to be assigned to more than one mature sequence. Subsequently, the precursor sequence of each entry is provided as input to the RNAfold tool of the ViennaRNA package (http://www.tbi.univie.ac.at/RNA). This procedure returns as a result the dot-bracket notation of the precursor, which is assigned as a fourth attribute to the corresponding entry. This step completes the phase of preprocessing, preparing the input for subsequent analysis. Now, the entries have four attributes in the following format:

(‘header’, ‘precursor’, ‘mature’, ‘dot-bracket’).

**Fig. 3:**
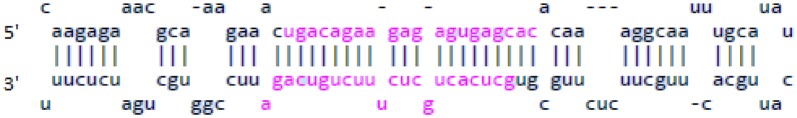
A pre-miRNA molecule with the sequences of miRNA and miRNA* highlighted in pink. Notice that the highlighted part is displaced by two nucleotides relatively to the one above it

These data are sufficient to provide a miRNA* sequence prediction. The procedure starts by making a list with all paired positions of the precursor molecule. The pairs are estimated based on the dot-bracket notation and stored in a data structure in the form of a dictionary. Then, the start and end positions of the mature sequences within the precursors are searched in this dictionary and their pair values are retained. Because of the property of the Dicer enzyme leaving two nucleotides hanging in each end during formation of the miRNA/miRNA* duplex (see Fig. 3), the previously mentioned positions are shifted by two. The new values will be the start and end positions of the miRNA* sequence within the precursor molecule, which is the final output of the analysis for each entry.

Some problems were identified during software testing. For example, there were occasions where one or both start and end positions of the miRNA sequence within the precursor were not paired and, therefore, they could not be matched with another position. The solution that was chosen is to include these unpaired positions in the pairs table, corresponding to a specific non-numerical value, in this occasion a wildcard character (*). This way, each position of the precursor sequence was represented in the pairs data structure either by a number indicating the pairing position or by a wildcard indicating that this position is unpaired. Consequently, each time the algorithm encounters an unpaired mature end, it shifts positions in the sequence until it finds a paired nucleotide, counting at the same time how many positions it has moved. After retrieving the miRNA* sequence, those missing nucleotides are added to the corresponding miRNA* end, so again the output is the correct opposite duplex strand.

## Results and discussion

To evaluate its predicting power, StarSeeker was applied to a dataset of precursor and already annotated mature sequences. This dataset consisted of all plant sequences available in miRBase [9]. The retrieved data were saved in two files, one containing 6992 precursor sequences and another with 8496 mature sequences.

**Fig. 4:**
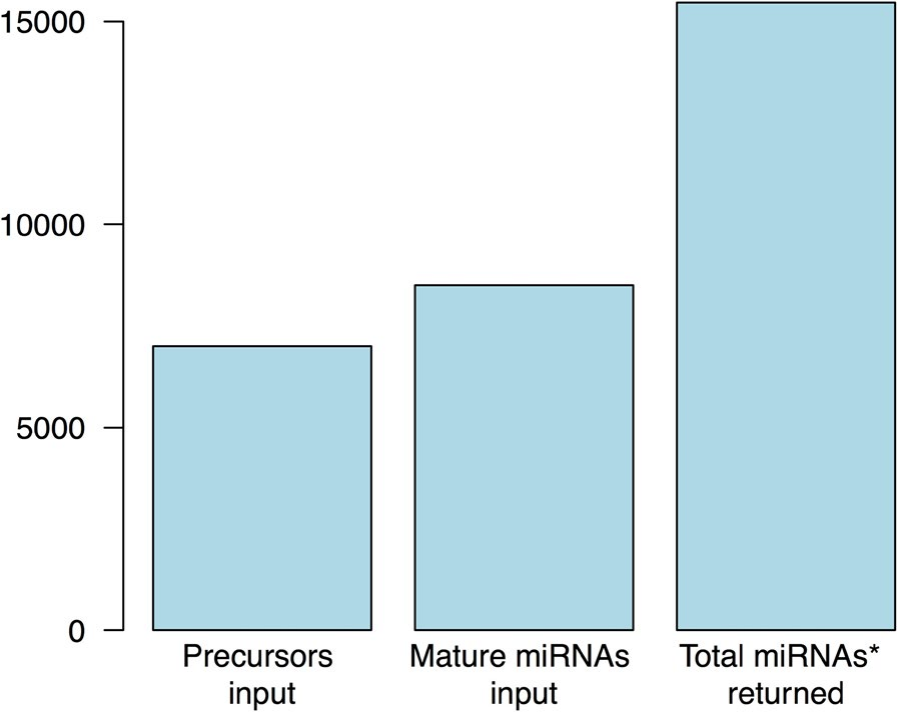
Input and results of running StarSeeker on all plant data contained in miRBase. The input files contained 6992 precursors and 8496 miRNAs. The output was 15,468 miRNAs*, because some mature sequences were matched to more than one precursor due to conserved genes and miRNA families among different species

These two files were used as input for analysis with StarSeeker. The program started by creating matches between mature and precursor sequences from the corresponding files. This process led to the formation of 192,816 pairs, because some miRNA families cause multiple matches within and between species. Then, all these pairs underwent analysis from the two functions of StarSeeker and a duplex solution was returned for each. In a next step, the duplicate entries were deleted and the final result was a non-redundant dataset of star sequences. The final size of this dataset was 15,468 sequences (Fig. 4).

Some errors that occurred during the analysis made the extraction of the star sequence not possible. For example, when each nucleotide of the mature miRNA within the precursor molecule was part of a loop (so the whole sequence was single stranded), the algorithm could not find a corresponding sequence on the opposite clone of the precursor hairpin structure. Also, when a large gap existed within the mature sequence, the opposite clone contained a bulge on these positions, which led to a very long star sequence, depending on the size of the gap. Each of these situations most probably represent events unlikely to occur naturally in the cell.

After running StarSeeker on the dataset, the .txt output file, which contained the 15,468 miRNA* sequences, was used to evaluate the reliability of the algorithm. Each star sequence was used as a query to search for matches within the initial mature miRNA source file. Some entries in miRBase contained annotations for both functional miRNA and miRNA* sequences. Therefore, the program must have found these annotated stars, using as template the mature miRNA. The evaluation analysis returned 2650 matches between source and output files. These sequences represent the annotated miRNAs* which were found by StarSeeker.

**Fig. 5:**
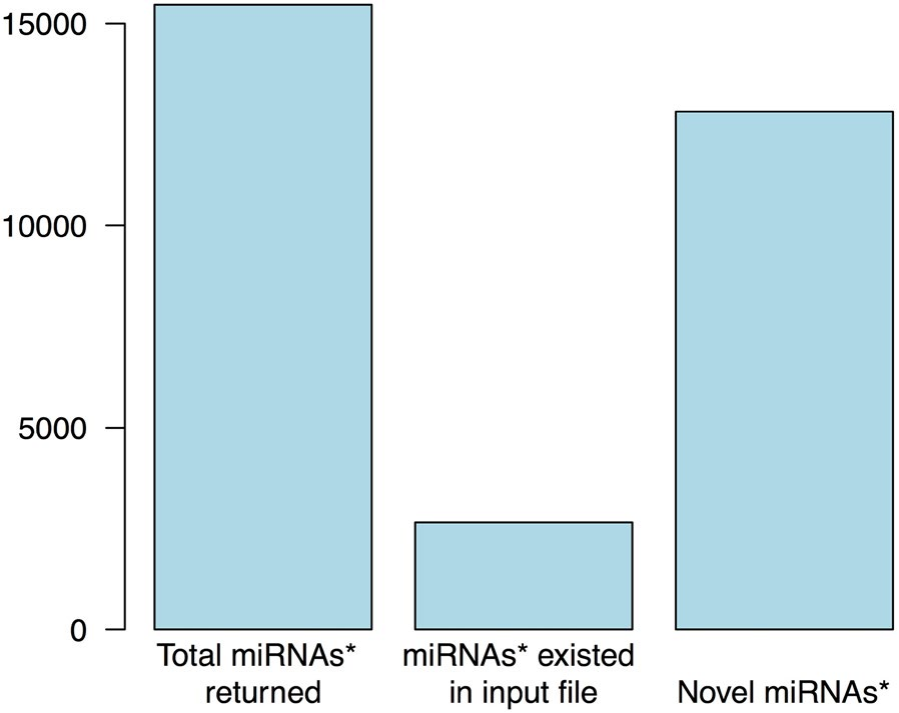
Evaluation of the output of running StarSeeker on plant sequences from miRBase. Of the 15,468 sequences that existed in the output file, 2650 were matched with the initial source mature miRNA data and 12,818 are considered novel miRNAs* and their existence can be verified by RNA-Seq experiments

**Fig. 6:**
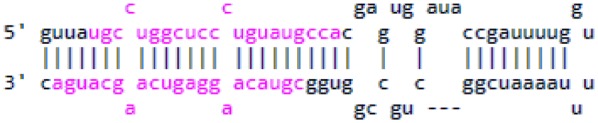
MiRBase entry of miR-160c from *Arabidopsis sp*. In this entry, the two annotated sequences of miRNA and miRNA* do not follow the biogenesis rule of two hanging nucleotides, as there are three nucleotides left in each end. This type of entries could not be verified during the evaluation process because the algorithm works only with the normal two-nucleotide ends duplexes

There are almost 1500 entries in miRBase that have both miRNA and miRNA* sequences annotated. Out of these 3000 sequences, the evaluation matched 2650 sequences (88.33%) (Fig. 5). The remaining 350 sequences could not be matched, mainly because they do not follow the two overhang nucleotides rule, as shown in Fig. 6.

The 12,818 sequences that were not matched to the source file are considered novel miRNA* sequences and they can be used as queries against RNA-Seq or other sequencing analysis data. Application of the StarSeeker tool can lead to interesting conclusions about plant miRNA-ome patterns under different stress conditions [6, 15, 16] (e.g. heat, absence of light) in various plant organisms.
